# Books and Magazines

**Published:** 1906-05

**Authors:** 


### Books and Magazines.

Modern Clinical Medicine. Translated from the German and edited under the supervision of Jules L. Salinger, M. D.

D. Appleton & Company are publishing at short intervals a series of books entitled “Modern Clinical Medicine,” a translation of “Die Deutsche Klinik,” a publication which is being brought out in parts in the German language. The articles upon the various diseases have been written by the most eminent men in Germany. Professors Leyden and Klemperer are the editors of the German work, and the contributors are such well-known authorities as Leube, Ewald, Boas, Baginsky, Liebermeister, Eichhorst, Strümpell, Curschmann, Jürgensen, Ehrlich, Grawitz, Binz, Nothnagel, Gerhardt, Loeffler, Senator, Krafft-Ebing, Hoffa, Ortner, Kaposi, and many others whose names are as familiar to you as the above mentioned.

It is the plan to have each volume edited by an eminent specialist in this country. Each of the volumes in this series can be bought separately.

---

DISEASES OF METABOLISM AND OF THE BLOOD, ANIMAL PARASITES, TOXICOLOGY. The second volume, just published, is edited with annotations by Dr. Richard C. Cabot, Assistant in Clinical Medicine, Harvard University Medical School, Boston, Mass. Price, cloth, $5.

This volume begins with a discussion of the quantitative analysis of disturbances of Metabolism, first relating to the consumption of food in the healthy, and second, to the food requirements of the sick, after which the Diseases of Metabolism are treated: Diabetes Mellitus, Diabetes Insipidus, Gout, Obesity, Myxedema, Addison's Disease, Acromegalia, Chronic Articular Rheumatism, and Pentosuria. The clinical side of these diseases is exhaustively gone into, and the treatment in all cases is thoroughly discussed.

Then follows a discussion of the Blood and Blood Examination, after which is taken up the Blood Diseases, as the Anemias, Chlorosis, Leukemia, Pseudo-Leukemia, and the Hemorrhagic Diatheses.

Over eighty pages are contributed to the question of Animal Parasites of man, and finally about twenty pages to the important poisons and their treatment. We have in English no work which gives the importance to the treatment of Constitutional Diseases as does this volume in Modern Clinical Medicine, and when we consider that it is the work of the best minds of Europe, backed up by an eminent editor, who has made many annotations to the articles under discussion, we may be sure we have a work which will command the attention and respect of every thoughtful American physician.

---

CASE TEACHING IN MEDICINE—Exercises in Diagnosis, Prognosis and Treatment of Actual Cases. By Richard C. Cabot, M. D. (Harvard). 8vo, 220 pp. with space for notes. Introduction and indexes. Price, $1.50.

There are 78 histories. Each is of special value in teaching and is followed by questions, answers, diagnosis, prognosis, treatment.

From the students' edition answers are omitted and space left to be filled in by the student himself. The book represents a plan that has been tried and is successful. Send for it. D. C. Heath & Co., Publishers, 120 Boylston Street, Boston.

---

SAUNDERS' QUESTION COMPENDS—ESSENTIALS OF GENITO-URINARY AND VENEREAL DISEASES. By Straling S. Wilcox, M. D., Professor of Genito-Urinary Diseases and Syphilology, Starling Medical College, Columbus, Ohio. 12mo of 313 pages, illustrated. Philadelphia and London: W. B. Saunders Co., 1906. Cloth, $1, net.

This little work is a worthy addition to Saunders' Question-Compend Series, a series that has reached a sale of over 265,000 copies. In this present work by Dr. Wilcox all genito-urinary and venereal diseases are fully detailed in the terse, direct language of question and answer, so that the student grasps immediately the point in question. Illustrations are freely used, adding much to the value of the book; and the large clinical experience of the author stamps it at once with accuracy and thoroughness. For the student there is none better; and the practitioner will find in it much that he is called upon every day to put into practice.

---

APPLETON'S MEDICAL AND SURGICAL PUBLICATIONS: ACCIDENT AND INJURY: THEIR RELATIONS TO DISEASES OF THE NERVOUS SYSTEM. By Pearce Bailey, A. M., M. D., Instructor in Neurology, Columbia University; Consulting Neurologist to St. Luke's, Roosevelt, Babies', and Orthopedic Hospitals, New York City. A new edition just out. Price not stated.

The original purpose of the author in this undertaking was to furnish a systematic description of the nervous affections which result from injury and fright, and which, under the generally familiar term of "the traumatic neuroses," exist independently of any as yet demonstrated lesion in the nervous system. These disorders are of sufficiently common occurrence to constitute a comprehensive chapter in internal medicine, and they have obtained a position of legal prominence from the frequency with which they are associated with questions of liability. Yet, in spite of their importance, that they are not always clearly understood by physicians generally is shown by the contradictory views expressed concerning them when they become the subjects of litigation.

While it can not be denied that the nature of traumatic neuroses is still in many respects obscure, sufficient evidence as to their causes and symptoms has now accumulated to permit them to be recognized as definite clinical types, and to be diagnosticated with reasonable accuracy. This evidence is, however, more or less inaccessible to general readers, existing chiefly as scattered monographs, most of which are in foreign languages. No single book has attempted, in recent years, to present in detail the enlarging views and the deductions from them concerning these disorders.

The necessary methods of examination of nervous cases are described in detail, so that it can at once be told, in the case of an expert witness giving an opinion in regard to any person alleged to be injured, whether his examination has been of a character to render the opinion of value.

Not the least interesting chapter is the one on Malingering, in which the various methods of feigning disease are described, and the means of unmasking simulators are given.

---

TRANSACTIONS OF THE MEDICAL ASSOCIATION OF ALABAMA. 1905. Brown Printing Co., Montgomery, Ala.

This Association is the State Board of Health, and was organized in 1847. The book is a handsome cloth-bound volume of 587 pages, and contains a full report of the proceedings, papers and discussions and the annual register of the county medical societies. Dr. L. C. Morris is secretary; Dr. C. C. Jones, of East Lake, president.

---

DISEASES OF THE EYE—A Handbook of Ophthalmic Practice. By G. E. deSchweinitz, M. D., Professor of Ophthalmology in the University of Pennsylvania. Fifth edition, revised and enlarged. Octavo of 894 pages, 313 text-cuts and 6 chromo-lithographic plates. Philadelphia and London: W. B. Saunders Co., 1906. Cloth, $5, net; half Morocco, $6, net.

Dr. deSchweinitz's work on the eye is so well known that anything but a mere mention of the new edition seems superfluous. The success it has achieved is readily accounted for if one but glance through its contents. In this edition, enlarged by the addition of new matter to the extent of some one hundred pages, there have been added, among other subjects, chapters on the following: X-Ray Treatment of Epithelioma, Xeroderma Pigmentosum; Purulent Conjunctivitis of Young Girls; Jequiritol and Jequiritol Serum; X-Ray Treatment of Trachoma; Infected Marginal Ulcer; Keratitis Punctata Syphilitica; Uveitis and Its Varieties; Eye-ground Lesions of Hereditary Syphilis; Macular Atrophy of the Retina; Worth's Amblyoscope; Stovain, Alypin; Motais' Operation for Ptosis; Kuhnt-Muller's Operation for Ectropion; Haab's Method for Foreign Bodies; and Sweet's X-Ray Method of Localizing Foreign Bodies. Other chapters have been rewritten. The excellence of the illustrative feature has been maintained and thirty-three additional text-cuts have been added. Dr. deSchweinitz's work was long ago recognized as an authority and this new edition goes a long way in strengthening its reputation, if any strengthening is needed.

---

Squibbs' Materia Medica: 1906 Edition.

This volume is intended to serve as a convenient, reliable and comprehensive hand-book and price list of all of Squibbs' preparations for the physician and the pharmacist.

---

Marriage in Free Society. By Edw. Carpenter, Stockham Publishing Co., Chicago. Pamphlet of 111 pages. Price not stated.

This seems to be one of a series of publications dealing with the sex problem, to which class belong "Toxology," or painless pregnancy and parturition. "The Lover's World," "Ethics of Marriage," and others, by Alice B. Stockham, M. D. "How to Live Forever," by Harry Gaze. "True Manhood the Secret of Power," by E. R. Shepherd, etc., etc. Full information can be had by writing to the publishers.

